# Regenerative Cardiology: Are We Close to Myocardial Repair?

**DOI:** 10.34172/jcvtr.33939

**Published:** 2026-06-30

**Authors:** Samad Ghaffari, Faezeh Tarighat, Seyedeh-Tarlan Mirzohreh, Neda Roshanravan, Sara Pahlavan

**Affiliations:** ^1^Cardiovascular Research Center, Tabriz University of Medical Sciences, Tabriz, Iran; ^2^Department of Stem Cells and Developmental Biology, Cell Science Research Center, Royan Institute for Stem Cell Biology and Technology, ACECR, Tehran, Iran

 Cardiovascular diseases are the major cause of death in the world, while myocardial infarction (MI) itself often results not only in the death of the heart muscle cells (cardiomyocytes) but also in progressive heart failure (HF).^1^ The heart, unlike other organs in the human body, has a limited capacity to regenerate, and effective repair from myocardial damage remains a great challenge in contemporary medicine.^2^ Regenerative cardiology is an innovative field of research focused on repairing damaged cardiac tissue using advanced techniques, including stem cell therapy, tissue engineering, genome editing, and extracellular vesicle treatments.^3^ Over the past two decades, this area has gained significant attention as a promising approach to restoring damaged heart tissue using these cutting-edge methods.^4^ Initial experiments had shown great promise for cardiac regeneration, but the clinical results have been mixed and, at best, were modest. However, molecular biology, bioengineering, and precision medicine have made recent strides that are changing the landscape of the discipline and offering hope for improved recovery of heart function.^5^

 Based on early evidence, in 2001, Orlic et al. published a ground-breaking study in Nature, which showed that the bone marrow-derived Lin⁻ c-kit⁺ hematopoietic cells, injected into infarcted murine myocardium, could transdifferentiate into cardiomyocytes and restore up to 68% of the infarcted ventricular wall within 9 days. This therapy resulted in a marked decrease in the left ventricular (LV) end-diastolic pressure and an improvement in myocardial performance, which suggested nearly full structural and functional recovery of the post-infarction heart.^4^ Going from experimental data to clinical use happened rather quickly and maybe too soon for adequate validation. Small clinical trials of intracoronary infusion of bone marrow-derived cells in patients after MI have already been conducted between 2002 and 2004. In particular, the BOOST trial demonstrated improvement in left ventricular ejection fraction (LVEF) of 6.7 percentage points at 6 months.^6^ The BOOST trial failed to show a significant increase in LVEF at 18-month follow-up, suggesting that it may have promoted early functional recovery instead of inducing long-term myocardial regeneration.^7^

 The first large randomized controlled trial in this area was the REPAIR-AMI trial (2006), which recruited 204 patients after ST-segment elevation myocardial infarction (STEMI) to receive either bone marrow mononuclear cells (BMMNCs) or a placebo intravenously. The study demonstrated a significant improvement in absolute LVEF at four months in the treatment group (5.5% vs. 3.0%, *P* = 0.01).^8^

 Over the years, numerous trials have been conducted in this field, and Table 1 summarizes some of the most important ones along with their results.

**Table 1:** Major Clinical Trials in Regenerative Cardiology and Their Key Outcomes

**Trial (Year)**	**n / Population**	**Primary Outcome**	**Key Finding**
BOOST (2004)^6^	60 / Post-MI	LVEF at 6 months	+ 6.7% LVEF; effect not sustained at 18 months
REPAIR-AMI (2006)^8^	204 / Post-STEMI	LVEF at 4 months	+ 5.5% LVEF; benefit only in LVEF < 49% subgroup
ASTAMI (2006)^9^	100 / Post-STEMI	LVEF at 6 months	No significant difference vs. control
FOCUS-CCTRN (2012)^10^	92 / Ischemic HF	LVESV, MPI, reversible defect	No improvement in any primary endpoint
C-CURE (2013)^11^	45 / Ischemic HF	LVEF at 2 years	+ 7.1% LVEF (from 27.5 ± 1.0% to 34.5 ± 1.1%)
BAMI (2019)^12^	375 / Post-STEMI	All-cause mortality	No difference at 2 years; first Phase 3 failure

Bone marrow transfer to enhance ST-elevation infarct regeneration: BOOST study; The Reinfusion of Enriched Progenitor Cells and Infarct Remodeling in Acute Myocardial Infarction (REPAIR-AMI) trial; The Autologous Stem-Cell Transplantation in Acute Myocardial Infarction (ASTAMI) study; First Mononuclear Cells Injected in the United States, conducted by the Cardiovascular Cell Therapy Research Network: FOCUS-CCTRN Trial; Cardiopoietic stem Cell therapy in heart failURE: C-CURE Trial; Heart failure: HF; Left ventricular ejection fraction: LVEF

 Over its 24-year history, regenerative cardiology has come a long way, and scientists have made substantial advances in myocardial repair. However, significant clinical hurdles remain before these advances can be translated into demonstrable therapeutic benefits. There are currently 23 clinical trials in cardiac regeneration underway worldwide, most of which are still in Phase 1 or Phase 2 (as of January 2024). No intervention has yet been shown, in a well-powered Phase 3 trial, to reduce mortality. The question of engraftment-related arrhythmias also remains unresolved. In addition, not all cardiac indications are FDA-approved, and no regenerative treatments are currently available.

## Conclusion

 As a general rule, current clinical experience suggests that myocardial regeneration is unlikely to be achieved. Stem cell therapies and other interventions have shown only short-term or mild improvement in heart function, and have not been shown to regenerate the heart tissue. These have been reinforced by recent large-scale syntheses of clinical trials. The recent meta-analysis of Muslem et al. (2025), which combined all stem cell trials from 2018, revealed again that the improvements in LVEF are still small and temporary, that mesenchymal stem cell (MSC) paracrine signaling decreases after 6 months, and that none of the trials have shown a decrease in all-cause mortality or HF hospitalization superior to statistical significance and with sufficient power.^13^

 Furthermore, Wulfse et al. (2025) conducted the most thorough systematic review of registered cardiac regenerative medicine clinical trials and concluded that only 1/3 of these trials had published results and that many trials were aborted or finished and not published. The authors also noted large study design variability and found that there was significant underreporting and inconsistent cell type, delivery method, dosing, timing, and outcome measures across studies, concluding that significant progress in the field is still needed due to these factors.^14^

 All these results together indicate that while the field of regenerative cardiology is a new and exciting one with great potential, it has yet to achieve the ultimate goal of true myocardial repair. What is needed for the future will likely be a transition from single modality strategies to more integrated strategies that involve cellular therapy, gene editing, and tissue engineering. The field is poised at a pivotal point currently: solid science has been established, but the prospect of clinical myocardial regeneration that will last beyond the surgery is still a work in progress.

## Competing Interests

 The authors declare that the research was conducted in the absence of any commercial or financial relationships that could be construed as a potential conflict of interest. Some of the authors are editorial board members of the Journal of Cardiovascular and Thoracic Research at the time of submission. This had no impact on the peer-review process and the final decision.

## Ethical Approval

 Not applicable.
